# Negative attitude towards the appearance: Connection with eating behavior and social anxiety

**DOI:** 10.1192/j.eurpsy.2021.1606

**Published:** 2021-08-13

**Authors:** E. Sedova, S. Kalina, Z. Gardanova

**Affiliations:** Psychological-social Department, Pirogov Russian National Research Medical University, Moscow, Russian Federation

**Keywords:** social anxiety, appearance, eating behavior

## Abstract

**Introduction:**

A negative attitude towards the body supposed to lead to eating disorders and to increase the level of social anxiety.

**Objectives:**

The research aim is to study the characteristics of eating behavior and social anxiety in women who have negative attitude towards their body.

**Methods:**

The following methods have been used: Multidimensional Body-Self Relations Questionnaire (MBSRQ); Eating Attitudes Test (EAT-26); Brief Fear of Negative Evaluation (BFNE); Iowa–Netherlands Comparison Orientation Measure (INCOM), Social avoidance and distress scale (SADS). The sample consists of 98 women in the age from 18 to 60 years belonged to three age groups: Group 1: N=41, mean age 21.0+3.1; Group 2: N=29, mean age 29,5+4,9; Group 3: N=28, mean age 47,5+12,5.

**Results:**

We have found out a statistically significant correlation between the negative attitude towards the body and the social anxiety. The more a woman dislike her appearance the higher is the level of social anxiety and the higher is the risk of eating disorders. It should be mentioned that all the negative tendencies are more prounouced in the youngest age group.

**Conclusions:**

The research results can be implemented when designing prevention programs. Such programs are extremely important for young women in the age from 18 to 24 years as they have the highest risk of forming an eatind disorder as well as the social anxiety disorder.

